# Blood-brain barrier permeability of gefitinib in patients with brain metastases from non-small-cell lung cancer before and during whole brain radiation therapy

**DOI:** 10.18632/oncotarget.3187

**Published:** 2015-02-06

**Authors:** Yin-duo Zeng, Hai Liao, Tao Qin, Li Zhang, Wei-dong Wei, Jian-zhong Liang, Fei Xu, Xiao-xiao Dinglin, Shu-xiang Ma, Li-kun Chen

**Affiliations:** ^1^ Department of Medical Oncology, Sun Yat-Sen University Cancer Center, State Key Laboratory of Oncology in South China, Collaborative Innovation Center for Cancer Medicine, Guangzhou 510060, China; ^2^ Lab of Phase I Clinical Study, Sun Yat-Sen University Cancer Center, State Key Laboratory of Oncology in South China, Collaborative Innovation Center for Cancer Medicine, Guangzhou 510060, China; ^3^ Department of Breast Oncology, Sun Yat-Sen University Cancer Center, State Key Laboratory of Oncology in South China, Collaborative Innovation Center for Cancer Medicine, Guangzhou 510060, China; ^4^ Department of Pathology, Sun Yat-Sen University Cancer Center, State Key Laboratory of Oncology in South China, Collaborative Innovation Center for Cancer Medicine, Guangzhou 510060, China; ^5^ Department of Medical Oncology, Sun Yat-Sen Memorial Hospital, Sun Yat-Sen University, Guangzhou 510120, China

**Keywords:** Non-small-cell lung cancer, brain metastasis, gefitinib, whole brain radiation therapy, blood brain barrier

## Abstract

**Introduction:**

To explore the ability of gefitinib to penetrate blood brain barrier (BBB) during whole brain radiation therapy (WBRT).

**Patients and Methods:**

Enrolled in this study were eligible patients who were diagnosed with BM from NSCLC. Gefitinib was given at 250 mg/day for 30 days, then concurrently with WBRT (40 Gy/20 F/4 w), followed by maintenance. Serial CSF and blood samples were collected on 30 day after gefitinib administration, and at the time of 10, 20, 30 and 40 Gy following WBRT. CSF and plasma samples of 13 patients without BM who were treated with gefitinib were collected as control. CSF and plasma gefitinib levels were measured by LC-MS/MS.

**Results:**

Fifteen BM patients completed gefitinib plus WBRT. The CSF-to-plasma ratio of gefitinib in patients with BM was higher than that in patients without BM (1.34% *vs.* 0.36%, *P* < 0.001). The CSF-to-plasma ratio of gefitinib increased with the increased dose of WBRT and reached the peak (1.87 ± 0.72%) at 30 Gy, which was significantly higher than that 1.34 ± 0.49% at 0 Gy (*P* = 0.01). The median time to progression of brain lesions and the median overall survival were 7.07 and 15.4 months, respectively.

**Conclusion:**

The BBB permeability of gefitinib increased in accordance with escalated dose of WBRT.

## INTRODUCTION

About 20–40% patients with non-small-cell lung cancer (NSCLC) develop brain metastasis (BM) [1–3]. The prognosis of BM from NSCLC is very poor with a median overall survival (OS) about 3–6 months in patients who received whole brain radiation therapy (WBRT). There are controversies over the role of systemic chemotherapy because of the limited ability of most potential agents to cross the blood–brain barrier (BBB) [4].

Studies [5] demonstrated that the integrity of BBB was disrupted in the presence of BM, and the BBB was leaky in a BM mouse model with tumors > 0.25 mm. In addition, BM-related blood vessels were dilated and contained many dividing endothelial cells. Qin et al [6] reported the image intensity was 22% higher in brain tumor area than normal brain area by collecting Count/pixel data in ^99M^Tc-GH imaging for a patient with BM which demonstrated the destructive effect of the BBB by brain tumor.

Epidermal growth factor receptor tyrosine kinase inhibitors (EGFR-TKIs) play an important treatment role for NSCLCpopulations worldwide. Gefitinib and erlotinib are oral EGFR-TKIs and have been approved in Asia for advanced NSCLC patients who have failed prior chemotherapy, or as first-line therapy for those with EGFR activating mutations [7–10]. Recently, gefitinib and erlotinib have been used for the treatment of patients with BM from NSCLC and reported effective against BM with a response rate (RR) of 10–58.3% and a disease control rate (DCR) of 22–77% in non-selected patients, bringing a median progression-free survival (PFS) of 3–9.7 months and a median OS of 8.3–18.9 months [11–17]. Whereas for patients harboring EGFR mutation, Park reported that the RR in brain was much higher as 83% [18]. Another study also reported the longer PFS for EGFR-mutated versus wide-type patients [19].

The role of EGFR-TKI with concurrent WBRT for NSCLC patients with BM is uncertain. A few studies showed that the combination of EGFR-TKI with concurrent WBRT has promising clinical activity. Ma et al [20] reported that the RR and DCR were 81% and 95% respectively in 21 NSCLC patients (EGFR non-selected) with BM treated by concurrent WBRT and gefitinib. Welsh et al [19] reported that the RR was 86% and the median OS was 11.8 months in patients treated with concurrent erlotinib and WBRT. There is no consensus regarding such treatment in NSCLC patients with BM. Future studies should focus on the role of EGFR-TKI with concurrent WBRT in patients with BM for more evidence.

Based on the efficacy of EGFR-TKIs, the cerebrospinal fluid (CSF) penetration of EGFR-TKIs is currently being investigated. Small-molecular-weight gefitinib may have the ability to penetrate the BBB [5, 21–22]. Studies reported that the CSF penetration rates of gefitinib and erlotinib were about 1–1.3% and 2.77–7%, respectively [22–29]. The CSF penetration rate of EGFR-TKI is relatively low.

Studies have found that BBB could be destroyed by radiation [21, 30–31]. Some studies [6, 32–34] reported that brain radiotherapy increased the penetration of anticancer drugs such as irinotecan, MTX and cisplatin into the CSF. Qin et al [33] reported that after irradiation of the brain with a dose of 20 Gy and intravenous administration of MTX to patients with brain tumors, the CSF-MTX concentration increased up to threefold. However, whether addition of WBRT could increase the permeability of gefitinib across the BBB remains unknown. In this prospective study, we evaluated the permeability of gefitinib across the BBB during WBRT in an attempt to obtain information about the efficacy and safety of gefitinib treatment with concurrent WBRT in NSCLC patients with BM.

## PATIENTS AND METHODS

The study was approved by the medical ethics committee of Sun Yat-Sen University Cancer Center (Guangzhou, China). Written informed consent was obtained from all patients before initiation of the study.

### Patients

The main inclusion criteria were pathologically confirmed NSCLC and clinically measurable brain metastases on magnetic resonance imaging (MRI) scans. Patients with 1–3 brain metastases were also eligible if they refused to receive neurosurgical or stereotactic radiosurgery (SRS).

Other eligibility criteria included age ≥ 18 years, ECOG PS of 0–3, a life expectancy > 3 months, and evidence of adequate hematologic and hepatic function. All patients were pretreated with at least one line of chemotherapy regimens or chemotherapy-naïve patients with EGFR mutations.

The main exclusion criteria included prior WBRT for BM, prior EGFR inhibitor therapy, uncontrolled active infection or other serious concomitant disorders, pregnant patients and patients with mental disorders. Also, those who had significant neurologic symptoms or signs were excluded, but those with asymptomatic or controlled symptomatic brain metastases by corticosteroids or mannitol were included.

In addition, in order to compare the baseline gefitinib CSF levels in non-BM patients with that in BM patients before WBRT, the study enrolled 13 patients without BM as control. They were pathologically confirmed NSCLC and in the absence of BM by MRI. The patients were pretreated with at least one line of chemotherapy or chemotherapy-naïve patients with EGFR mutations and appropriate for gefitinib treatment.

### Treatment

**Figure 1:**
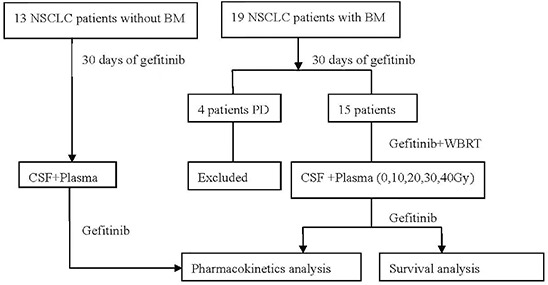
Disposition of patients (CONSORT diagram)

For NSCLC patients with BM, Gefitinib was administered orally at a daily dose of 250 mg for 30 days, continued concurrently with WBRT (40 Gy/20 F) for 4 weeks, and then maintained at the same dosage before occurrence of severe or intolerable toxicity, disease progression, or death. For NSCLC patients without BM, gefitinib was administered orally at a daily dose of 250 mg before occurrence of severe or intolerable toxicity, disease progression, or death. Tumor tissue specimens were collected to detect EGFR mutations by DNA direct sequencing. The study design is shown in Figure 1.

For patients with BM, serial CSF and blood samples were collected on day 30 after gefitinib administration before WBRT, and at the time of 10, 20, 30 and 40 Gy following WBRT. For patients without BM, blood and CSF samples only on day 30 after gefitinib administration were collected.

### Pharmacokinetic data

Gefitinib as primary standard and vandetanib as internal standard were supplied by AstraZeneca.

### Blood and CSF sampling and detection of gefitinib levels

Plasma and CSF concentrations of gefitinib were determined by a validated high-performance liquid chromatographic method (HPLC) with tandem mass spectrometric detection (LC/MS/MS), as described previously [35]. Plasma and CSF were isolated by centrifugation at 5000 rpm at 4°C for 5 min. Plasma and CSF analytes were extracted by liquid-liquid extraction using methyl tert-butyl ether (MTBE). Supernatants were evaporated to dryness and reconstituted with mobile phase. The samples were injected onto the liquid chromatography/mass spectrometry (LC-MS) system, chromatographed with an Inertsil ODS3 column (2.1*150 mm*3 um) at 25°C column temperature, and detected using a PE Sciex API 2000 triple quadrupole mass spectrometer with a turbo ionspray source interfaced to an Agilent-1200 HPLC system. The mobile phase consisted of 0.02 M ammonium acetate-acetonitrile (70:30, v/v, pH = 3). The flow rate was set at 0.25 ml/min, and all separations were carried out at 25°C. The running time of each sample was 6 min. Samples were quantified by the internal standard reference method in the MRM mode by monitoring the transition m/z 447.2→128.1 for the analyte gefitinib and m/z 475.6→112.0 for the internal standard vandetanib. Standard curves were linear (r2 > 0.99) over the range of 1–600 ng/ml. The lower limit of quantification (LLOQ) of the method was 1 ng/ml. The extraction recovery for vandetanib in plasma at 50 ng/ml was 80.62%. For detection of gefitinib in plasma samples, the extraction recovery of gefitinib at 1, 3, 300, 480 ng/ml was found in the range of 74.47–84.52%. The intra- and inter-batch precisions (RSD %) and the intra- and inter-batch accuracies were within 15%.

### Efficacy and safety analyses

Baseline assessment was performed within 2 weeks before gefitinib treatment, including medical history, physical examination, hematology and biochemistry test, chest CT and brain MRI. Chest CT scan and brain MRI were performed 30 days after gefitinib treatment and completion of WBRT, and then at 2-month intervals before death or loss to follow-up. Tumor response was evaluated according to the RECIST version 1.0 [36]. Toxicity evaluation was based on the National Cancer Institute Common Toxicity Criteria (NCICTC) version 3.0 and assessed monthly.

### Statistical methods

PFS was calculated from the initiation of gefitinib administration to disease progression or death from any cause. Time to progression (TTP) was calculated from the initiation of gefitinib administration to disease progression. OS was calculated from the initiation of gefitinib administration to death from any cause. This study had an 90% power in order to detect a 1% higher in mean CSF-to-plasma ratio of gefitinib after WBRT compared with that before WBRT, with Standard deviation 1% and 20% drop rate at a two-sided significance level of 0.05.(see Supplementary Material File). Two independent sample *t*-test was used to determine difference of CSF-to-plasma of gefitinib between BM and no BM. Paired sample *t*-test were used to determine the gefetinib concentration of different durations. The relationships between CSF and serum concentrations of gefetinib were determined by linear regression. The survival curves were generated using the Kaplan–Meier method. Univariate analysis of patient characteristics and tumor responses was conducted by Pearson chi-square test or the Fisher exact test. Multivariate analysis was evaluated using a logistic regression model to predict the clinical response to the treatment regimen. The following variables were included: gender, age, performance status, smoking history, number and size of brain metastases, EGFR mutation status, and extracranial metastases. The Cox regression method was used to identify the most important independent prognostic factors and estimate the hazard ratio. All tests and confidence intervals (CIs) were two sided and a significance level was 0.05. Statistical analyses were performed by using SPSS software, Version 13.0.

## RESULTS

### Baseline characteristics

From October 2009 to March 2011, 19 NSCLC patients with BM were enrolled, of whom four patients progressed 30 days after gefitinib treatment, and the remaining 15 patients received gefitinib with concurrent WBRT. The baseline characteristics of these 15 patients are listed in Table 1. All the 15 patients underwent EGFR testing, finding 6 patients who had EGFR mutations, including 5 exon 19 deletions and 1 exon 21 L858R point mutations.

**Table 1:** Patient characteristics (*n* = 15)

Characteristics	*N*	*%*
Age (yrs)		
Median (yrs)	52	
Range (yrs)	20–72	
≤65	14	93.33
>65	1	6.67
Gender		
Male	7	46.67
Female	8	53.33
Pathology		
Adenocarcinoma	13	86.67
Adenosquamous carcinoma	2	13.33
Performance status		
0–2	13	86.67
3	2	13.33
Smoking		
Never or light smoker	9	60
Heavy smoker	6	40
Initial diagnosis of BM		
Yes	7	46.67
No	8	53.33
No. of brain metastases		
Single	6	40
Multiple	9	60
Size of brain metastases (mm)		
≤20	14	93.33
>20	1	6.67
CNS symptoms		
Yes	5	33.33
No	10	66.67
EGFR mutations		
Negative	9	60
Positive	6	40
Organs of extracranial metastases		
Yes	12	80
No	3	20
No.of prior chemotherapy		
0	3	20
1	6	40
≥2	6	40
Prior thoracic irradiation		
Yes	3	20
No	12	80
RPA grouping		
1	5	33.33
2	9	60
3	1	6.67
Prior stereotactic radiosurgery	1	6.67

Abbreviation: BM, brain metastasis; CNS, central neurology system; EGFR, epidermal growth factor receptor; RPA, recursive partitioning analysis.

### Pharmacokinetic results

Samples from the 13 patients without BM and the 15 patients with BM were collected and analyzed.

**Figure 2:**
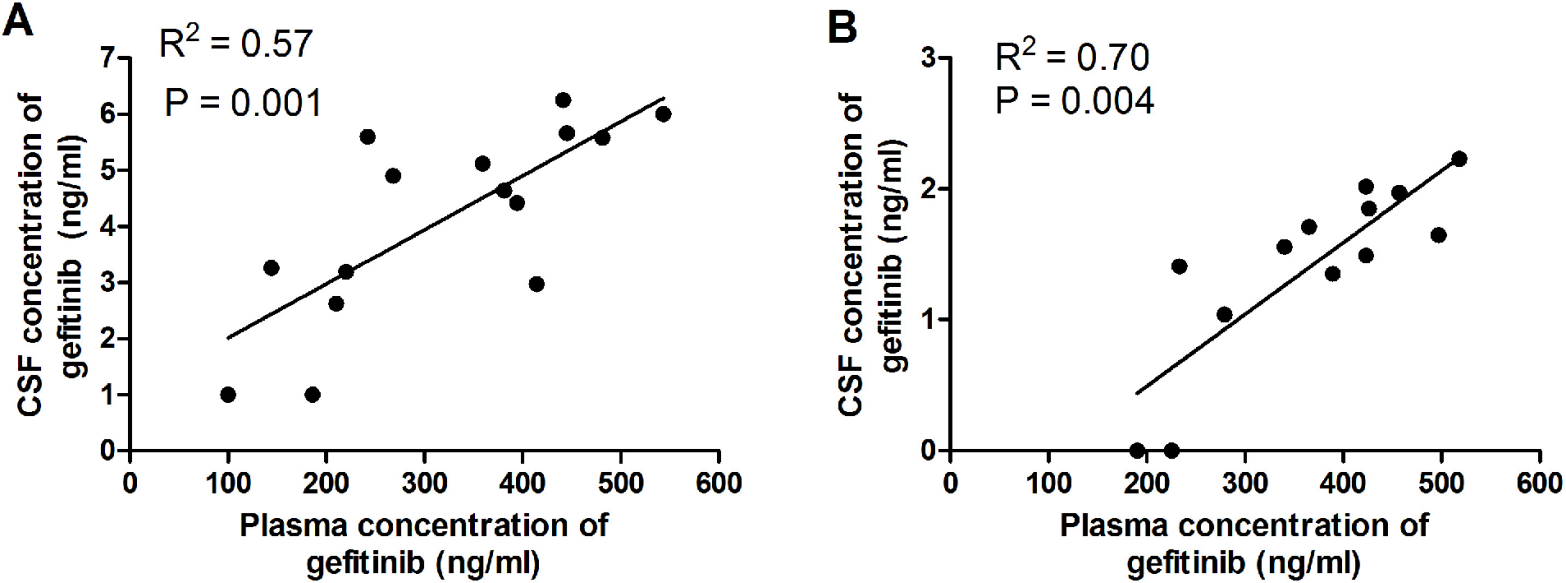
**(A)** Correlation between plasma and CSF concentrations of gefitinib in 15 patients with BM. A good correlation (R^2^ = 0.57) was demonstrated (*P* = 0.001). **(B)** Correlation between plasma and CSF concentrations of gefitinib in 13 patients without BM. A good correlation (R^2^ = 0.70) was demonstrated (*P* = 0.004).

**Figure 3:**
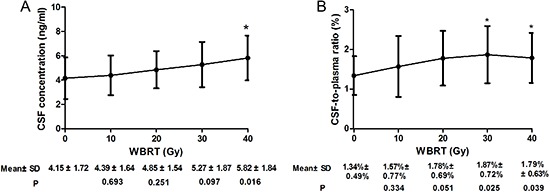
Gefitinib CSF concentration (A) and CSF-to-plasma ratio of gefitinib concentration (B) during WBRT compared with that of baseline (0 Gy) *P < 0.05.

The plasma concentrations of gefitinib were similar between patients with and without BM. Both the mean CSF concentration of gefitinib and the CSF-to-plasma ratio of gefitinib in patients with BM were significantly higher than those in patients without BM (Table 2, *P* < 0.001). A good correlation (R2 = 0.57) between plasma and CSF concentrations of gefitinib in 15 patients with BM before WBRT was demonstrated (*P* = 0.001, Figure 2A). Similarly, a good correlation (R2=0.70) between plasma and CSF concentrations of gefitinib was demonstrated in 13 patients without BM (*P* = 0.004, Figure 2B). The CSF concentration of gefitinib and the CSF-to-plasma ratio increased with escalation of the WBRT dose (Figure 3A, 3B). The mean CSF concentration of gefitinib at 30 and 40 Gy was statistically higher than that at 0 Gy. The CSF-to-plasma ratio of gefitinib reached the peak (1.87 ± 0.72%) at 30 Gy, which was significantly higher than 1.34 ± 0.49% at 0 Gy (*P* = 0.01). Pharmacokinetic analysis indicated that the addition of WBRT enhanced gefitinib's ability of penetration in CSF.

**Table 2:** The comparison of gefitinib concentration in CSF and blood between patients with BM before WBRT and patients without BM

Body fluid	Concentration (ng/ml)		*P* value
	Non-BM group (*n* = 13)	BM group (*n* = 15)	
CSF	1.41 ± 0.7	4.15 ± 1.72	<0.001
Blood	366.54 ± 106.44	321.87 ± 134.60	0.344
Ratio (%)	0.36 ± 0.18	1.34 ± 0.49	<0.001

Abbreviation: CSF: cerebrospinal fluid; BM, brain metastases

The mean CSF concentration of gefitinib at 20–40 Gy (4.85–5.82 ng/ml) reached IC50 (4.46–8.9 ng/mL /10–20 nM/L) for EGFR mutant NSCLC cell lines [37].

The number (single *vs*. multiple) and size (≤20 mm *vs.* > 20 mm) of brain lesions did not significantly affect the permeability of gefitinib in patients with BM from NSCLC. The EGFR mutation status was not related to the permeability of the BBB to gefitinib.

### Efficacy outcomes

Five patients treated with gefitinib and concurrent WBRT were alive at the time of this analysis, and the median follow-up time was 15.4 months (range 4.1–28.23 months). The median PFS and OS were 6.17 and 15.40 months, respectively. The median TTP for intracranial and lung lesions were both 7.07 months. Tumor responses of extracranial lesions and brain metastases were similar (Table 3).

**Table 3:** Response and survival of 15 patients with BM from NSCLC treated by gefitinib plus WBRT

	*N*	*%*
Brain metastases		
RR (%)	6	40
DCR (%)	13	86.67
TTP (months)	7.07(95%CI:3.24–10.90)	
Primary thoracic lesions		
RR (%)	6	40
DCR (%)	12	80
Median TTP (months)	7.07(95%CI:2.14–11.99)	
Overall		
RR (%)	6	40
DCR (%)	12	80
Median PFS (months)	6.17 (95%CI:1.50–10.84)	
Median OS (months)	15.40 (95%CI:11.33–19.47)	
Rate at 1-yr (%)		66.6
Rate at 2-yr (%)		38.9

Abbreviation: BM, brain metastasis; WBRT, whole brain radiation therapy; RR: response rate; DCR: disease control rate; TTP, time to progression; PFS: progression-free survival; OS: overall survival; 95%CI, 95% confidential internal.

**Figure 4:**
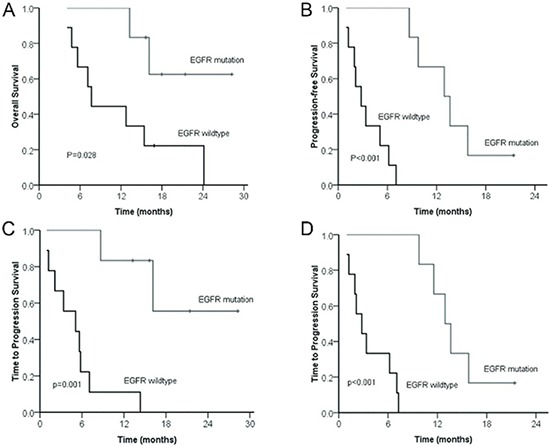
Comparison of overall survival (A) and progression-free survival (B) between patients with BM from NSCLC according to EGFR mutation status Comparison of time to progression of brain lesions **(C)** and lung lesions **(D)** from patients with BM from NSCLC according to EGFR mutation status.

The treatment response and survival analysis of the 15 patients according to the EGFR mutation status were shown in Table 3. The median PFS and OS in patients with EGFR mutations were significantly longer, compared with patients with EGFR wild type (*P* < 0.05, Figure 4A, 4B). The median TTP of either brain lesions or primary lung lesions was significantly longer in EGFR mutant patients (*P* < 0.05) (Figure 4C, 4D). In addition, the RR of BM was higher in patients with EGFR mutant disease than that in patients with wild-type disease (83.33% *vs*.11.11%) (Table 4).

**Table 4:** Treatment response of patients with BM from NSCLC treated by gefitinib plus WBRT according to EGFR mutation status

Response	EGFR		*P* value
	Wild-type (*n* = 9)	Mutant (*n* = 6)	
Brain metastases			
RR (%)	1(11.11%)	5(83.33%)	0.01
DCR (%)	7(77.78%)	6(100%)	0.49
Median TTP (months)	5.10(95%CI: 0.04–10.16)	—	0.001
Primary thoracic lesions			
RR (%)	1(11.11%)	5(83.33%)	0.01
DCR (%)	6(66.67%)	6(100%)	0.23
Median TTP (months)	2.80(95%CI:0.85–4.74)	12.93(95%CI:10.45–15.41)	<0.001
Overall			
RR (%)	1(11.1%)	5(83.33%)	0.01
DCR (%)	6(66.7%)	6(100%)	0.23
Median OS (months)	7.67(95%CI: 6.11–9.23)	—	0.03
Median PFS (months)	2.80(95%CI: 0.85–4.75)	12.93(95%CI:8.29–17.57)	<0.001

Abbreviation: BM, brain metastasis; WBRT, whole brain radiation therapy; RR: response rate; DCR: disease control rate; TTP, time to progression; OS: overall survival; PFS: progression-free survival; 95%CI, 95% confidential internal; —: not yet reached.

### Toxicity and safety

All 15 patients were included in the toxicity analysis. The reported adverse events (AEs) are summarized in Table 5. The most common AEs reported were rash (53.3%, 8/15) outside the radiation field, acne (33.3%, 5/15), radiation field dermatitis (20%, 3/15), paronychia (20%, 3/15), pruritus (20%, 3/15), fatigue (26.7%, 4/15), diarrhea (33.3%, 5/15), and vomiting (20%, 3/15). We saw no cases of radiation enhancing the gefitinib-related rash in the portal treatment area. All toxicities were grade 1 or 2. The reported neurotoxicities during the combined treatment were headache (20%), dizziness (6.7%), memory impairment (6.7%), and hydrocephalus (6.7%). We saw no cases of leukoencephalopathy and cognitive disturbance. No statistically significant differences were found between gefitinib-WBRT group and the historical WBRT group in the proportion of patients with evidence of neurotoxicity. Patients had no treatment related ocular symptoms.

**Table 5:** Treatment-related toxicities during gefitinib with concurrent WBRT

Adverse event	Grade 1/2, *n*	Grade 3/4, *n*	Total, *n* (%)
Rash	8	0	8 (53.3)
Acne	5	0	5 (33.3)
Radiation dermatitis	3	0	3 (20)
Dry skin	2	0	2 (13.3)
Pruritus	3	0	3 (20)
Paronychia	3	0	3 (20)
Vomiting	3	0	3 (20)
Diarrhea	5	0	5 (33.3)
Fatigue	4	0	4 (26.7)
ALT elevation	1	0	1 (6.7)
Pneumonia	0	0	0
Headache	3	0	3 (20)
Cognitive disturbance	0	0	0
Confusion	0	0	0
Dizziness	1	0	1 (6.7)
Hydrocephalus	1	0	1 (6.7)
Leukoencephalopathy	0	0	0
Memory impairment	1	0	1 (6.7)
Seizure	0	0	0

## DISCUSSION

Brain metastases are associated with poor prognosis, for which there is no effective treatment at present. WBRT is a standard treatment for BM. The low CSF penetration of chemotherapeutic drugs remains a significant factor contributing to poor therapeutic outcomes for BM patients. Small-molecular-weight (446.9 daltons) gefitinib may have the ability to penetrate the BBB [5, 21–22]. Some studies [21] reported that BM and WBRT could disrupt the BBB. To the best of our knowledge, the effect of WBRT on gefitinib permeability across the BBB has not been previously reported. The major objective of our study was to evaluate whether WBRT could affect BBB permeability of gefitinib in BM patients.

For years, brain metastasis has been considered to increase the permeability of the BBB [38]. Our study presented evidence that gefitinib could only reach a rather low level in patients without BM, with a CSF-to-plasma gefitinib ratio about 0.36% ± 0.18%. Our study showed that CSF level of gefitinib was 4.15 ng/ml and the CSF-to-plasma ratio of gefitinib before WBRT was only 1.34%, which is similar to that reported in other studies [22–24, 39]. The CSF-to-plasma gefitinib ratio in patients with BM before WBRT was significantly higher than that in patients without BM. Although BM could disrupt the BBB, the CSF penetration ability of gefitinib remains low.

WBRT can disrupt the BBB. d'Avella et al [30, 32] reported that WBRT (40 Gy/20 F) induced changes in BBB function with the significant increase of transport of ^14^C-alpha-aminoisobutyric acid across the BBB in the cerebral cortex 15 days after WBRT. Qin et al [6, 33] observed that radiotherapy enhanced the destruction of the BBB and the degree of the destructive effect on the BBB in the irradiated normal area directly proportional to the radiation dose, and the ratio of MTX concentration in blood and CSF increased by 1.43 fold after 40 Gy WBRT. Some studies [33–34] reported that brain radiation could increase the penetration of anticancer drugs such as irinotecan and cisplatin into the CSF. However, there are no current data to support the effect of WBRT on gefitinib permeability across the BBB in patients with BM from advanced NSCLC. Our study also showed that the CSF-to-plasma ratio of gefitinib increased with the increased dose of WBRT and reached the peak (1.87 ± 0.72%) at 30 Gy, which is significantly higher than 1.34 ± 0.49% at 0 Gy (*P* = 0.01). In addition, Gow et al [40] reported that the administration of EGFR TKI during WBRT conferred radiosensitivity in brain metastasis of lung adenocarcinoma. WBRT may enhance the efficacy of gefitinib for BM patients.

The mean CSF concentration of gefitinib at 20–40 Gy was 4.85–5.82 ng/mL, reaching the IC50 of gefitinib *in vitro* of EGFR-mutant cell lines [37]. This may explain the high disease control of brain lesions (RR 83.33%; DCR 100%) in EGFR mutant patients with BM. Therefore, BM patients with EGFR mutations could benefit more from gefitinib. In addition, we also found that the CSF concentration of gefitinib was well correlated with its plasma concentration. Some studies [22, 41] reported that administration of high-dose EGFR-TKIs could achieve a higher CSF concentration and clinical efficacy as compared with standard dosing. One study [22] reported that the CSF concentration of gefitinib increased with the increased dose of gefitinib, ranged from 6.2 to 18 nM at a 500-mg dose, and reached 42 nM at a 1,000-mg dose in patients with leptomeningeal metastasis. Clarke et al [41] reported that patients with NSCLC leptomeningeal metastases treated with 1500 mg erlotinib weekly demonstrated a peak plasma concentration of 11,300 nM with a concurrent CSF concentration of 130 nM exceeding the IC50. Therefore, high-dose pulsatile EGFR-TKIs may be an alternative strategy to treat central nervous system (CNS) metastases from NSCLC with EGFR wild-type. Grommes et al [42] reported that pulsatile erlotinob at approximately 1500 mg per week was safe and had activity in patients with CNS diseases from EGFR mutant NSCLC even when systemic resistance had developed and been confirmed.

Our study showed that penetration of gefitinib into CSF was facilitated under the condition of BM- and WBRT-induced BBB disruption, which supports the beneficial effect of using gefitinib with concurrent WBRT in patients with BM. However, previous studies on the efficacy of EGFR-TKIs with concurrent WBRT have yielded conflicting results. Pesce et al [43] reported that the median OS in their 16 patients with BM treated with gefitinib and concurrent WBRT was 6.3 months, showing no clinical benefit compared with historical controls receiving gefitinib alone [12, 14, 17, 44]. Welsh et al [19] reported that in their 40 patients treated with erlotinib plus WBRT, the median OS for those with EGFR wild type and EGFR mutations were 9.3 and 19.1 months, respectively. Despite clinically significant findings, it still has some limitations. The relatively small number of patients recruited should be considered when interpreting the results. In addition, EGFR status was confirmed using samples from lung lesions and not with intracranial lesions. Further studies are needed to confirm the clinical benefit of EGFR-TKIs and concurrent WBRT in patients with BM.

Pharmacokinetically, our study supports the combination of EGFR-TKIs with concurrent WBRT in treating patients with BM, especially for patients with activating EGFR mutations. Further studies could be made to compare concurrent and sequential therapy of gefitinib and WBRT. It remains unknown whether EGFR-TKI with concurrent WBRT is superior to EGFR-TKI alone in NSCLC with BM, especially for patients with activating EGFR mutations. In addition, CSF level of gefitinib may not be the same as that in patients with brain lesions, and disruption of the BBB in BM may be more severe than that in the surrounding tissues. It seems more reasonable to study the drug concentration within the brain tumor in study of CNS pharmacokinetics of anticancer therapies.

## EXPLANATION FOR SAMPLE SIZE STATISTICS


