# Supplementary material for: Blood-brain barrier permeability of gefitinib in patients with brain metastases from non-small-cell lung cancer before and during whole brain radiation therapy
Source: Oncotarget. 2015 Feb 6;6(10):8366–76. doi: 10.18632/oncotarget.3187 (PMC4480758; doi:10.18632/oncotarget.3187)
Supplement: Supplementary file 1 [file oncotarget-06-8366-s001.pdf]

EXPLANATION FOR SAMPLE SIZE STATISTICS

The study was designed to explore whether BM improved CSF-to-plasma ratio of gefitinib and whether concurrent WBRT elevated CSF-to-plasma ratio of gefitinib for patients with BM. We finally selected the larger sample size to enroll candidates after calculating sample size addressing the two questions above by using two-sample *T* test for comparing BBB permeability of non-BM with BM and paired-sample *t* test for analyzing WBRT induced changes of BBB respectively. The hypothesis and methods used to calculate sample size were shown respectively as followed:

1. To explore whether BM improved the CSF-to-plasma ratio of gefitinib for patients with NSCLC: Though a recent study conducted by Zhao J and colleagues showed that CSF-plasma concentration ratio of gefitinib were 0.9% and 1.3% in NSCLC without or with BM, respectively [Jing Zhao et al. Clinical Lung Cancer, 2012], no data on CSF concentration of gefitinib in patients was available when our study was designed. In our previous study, we found that CSF concentration of DDP was elevated twice in patients with BM compared with those patients without BM (0.33 mg/L, for no BM patients and 1.02 mg/L for BM patients [Chen LK et al. Chinese Journal of cancer. 2001, 20(3):901–903]. The CSF-to-plasma ratio of gefitinib was 0.68% and 1.05% for patients with BM by two case reports [Tatsuro F et al. Tohoku J. Exp. Med., 2008; David MJ et al. J Clin Oncol, 2006]. Preclinical study showed that brain concentration to lung concentration ratio of gefitnib was 2% [Mckillop D, et al. Xenobiotica. 2004], but no study reported the extent of gefitinib concentration escalation of BM versus no BM.

Based on that, we did pre-experiment to obtain the CSF-to-plasma ratio of gefitinib of patient without and with BM. We tested CSF and plasma concentration of one NSCLC patient administering gefitinib without and with BM, respectively. Both of them had administered a period of 30 days gefitinib before CSF and plasma detection by high-performance liquid chromatographic method. The CSF concentration of gefitinib of patient without BM and patient with BM were 1.54 ng/ml and 3.03 ng/ml, respectively. The plasma concentration of gefitinib were 324.32 ng/ml and 316.63 ng/ml, respectively. The CSF-to-plasma ratio of gefitinib were 0.46% and 0.96%, respectively. We conservatively supposed the improvement in CSF-to-plasma ratio of patients with BM is 0.5% compared with that of patients without BM. Two-sample *T* test for independent samples method was adopted supposed assuming that the difference of CSF-to-plasma ratio of gefitinib in two sample were adequately normally distributed.

The formula as followed:

$$n = (q_1^{-1}+q_2^{-1})\,(t_\alpha+t_\beta)^2S^2/\delta^2$$

The calculation of the number of patients required in this study was based on assumptions:

One-sided two-samples *t* test for independent samples

$q_1 = q_2 = 0.5$

0.05 alpha with 80% power

CSF-to-plasma ratio of gefitinib without BM: 0.5%

Mean CSF-to-plasma ratio of gefitinib with BM: 1 %

Standard deviation = 0.5%

Based on these assumption a total of 13 patients were needed in each group.

2. To explore the effect of WBRT on CSF/plasma ratio of gefitinib in patients with BM: Unfortunately, till now no study had reported the change of gefitinib concentration during WBRT. Our previous study demonstrated that the BBB is gradually opened by WBRT. The mean CSF concentration of DDP after 30Gy WBRT was two to three times higher than that before WBRT [LK Chen et al. Chinese Journal of cancer, 2001; DX QIN et al. Am J Clin Oncol, 1997]. Gefitinib was a small molecular weight EGFR-TKI which was more likely to penetrate BBB than chemotherapy agents. Therefore, we supposed mean CSF-to-plasma ratio of gefitinib with BM before WBRT was 1% based on previous reports and our testing results above. We made a hypothesis that CSF-to-plasma ratio after WBRT was 2%. Assuming that the difference of CSF-to-plasma ratio of gefitinib under normal distribution, paired-sample *t* test was adopted. The formula as follow:

$$n = [(t_{\alpha/2}+t_\beta)S/\delta]^2$$

The calculation of the number of patients required in this study was based on assumptions:

Two-sided paired-sample *t* test for single sample

0.05 alpha with 90% power

Mean CSF-to-plasma ratio of gefitinib before WBRT: 1%

Mean CSF-to-plasma ratio of gefitinib after WBRT: 2%

Standard deviation = 1%

Dropout rate: 20% patients may be progression

Based on these assumption a sample size of approximately 15 patients was required.

In order to test the hypothesis, 15 patients were needed to conduct this study.
